# Changing in Larch Sapwood Extractives Due to Distinct Ionizing Radiation Sources

**DOI:** 10.3390/ma14071613

**Published:** 2021-03-26

**Authors:** Thomas Schnabel, Marius Cătălin Barbu, Eugenia Mariana Tudor, Alexander Petutschnigg

**Affiliations:** 1 Department Forest Products Technology and Timber Constructions, Salzburg University of Applied Sciences, Markt 136a, 5431 Kuchl, Austria; marius.barbu@fh-salzburg.ac.at (M.C.B.); Eugenia.tudor@fh-salzburg.ac.at (E.M.T.); alexander.petutschnigg@fh-salzburg.ac.at (A.P.); 2 Faculty of Furniture Design and Wood Engineering, Transilvania University of Brasov, B-dul. Eroilor nr. 29, 500036 Brasov, Romania; 3 Salzburg Center for Smart Materials, c/o Department of Chemistry and Physics of Materials, Paris Lodron University of Salzburg, Jakob-Harringer-Strasse 2A, 5020 Salzburg, Austria; 4 Department of Material Sciences and Process Engineering, University of Natural Resources and Life Sciences (BOKU), Konrad Lorenz-Straße 24, 3340 Tulln, Austria

**Keywords:** EBI, γ-ray, GC-MS, FT-IR, larch sapwood, wood extractives

## Abstract

Wood extractives have an influence on different material properties. This study deals with the changes in wood extractives of larch sapwood due to two different low doses of energy irradiations. Electron beam irradiation (EBI) and γ-ray irradiation treatments were done by using two industrial processes. After the different modifications the extractions were performed with an accelerated solvent extractor (ASE) using hexane and acetone/water. The qualitative and quantitative chemical differences of irradiated larch sapwood samples were analysed using gas chromatography–mass spectrometry (GC-MS) and Fourier-transform infrared spectroscopy (FT-IR) vibrational spectroscopy methods. The yields of the quantitative extractions decreased due to the two different irradiation processes. While the compounds extracted with nonpolar solvent from wood were reduced, the number of compounds with polar functionalities increased based on the oxidation process. Quantitatively, resin acids and polyphenols were highly affected when exposed to the two irradiation sources, leading to significant changes (up, down) in their relative amount. Furthermore, two new substances were found in the extracts of larch sapwood samples after EBI or γ-ray treatments. New insight into the different effects of larch sapwood and wood extractives by EBI and γ-ray was gained in this study.

## 1. Introduction

The effects of irradiation of different wood polymers have been studied since the early 1960s [1]. The irradiated polymers undergo various complex changes in chemistry [2,3,4,5]. By varying the irradiation doses and radiation sources, the material properties can be differently influenced, and therefore can be used for different applications connected to the functionalisation of cellulose and lignin [6,7,8]. The wood extractives may influence these chemical changes caused by irradiation. This study deals with the changes in wood extractives of larch sapwood due to low doses of irradiation.

In general, two different reasons for irradiation treatments can be distinguished. On the one hand, ionising radiation was investigated for a pre-treatment pulping process to separate the cellulose and lignin with a low accumulated dose and less chemicals compared to conventional processes [5,9]. A few efforts have been made to force the degradation of lignin without a cross-linking process [2]. 

On the other hand, the irradiation has been used to functionalise different properties for the enhancement of wood [6,10]. After a low dose of electron beam irradiation of wood, the weathering effects were reduced and the colour stabilisation was improved [7]. Condensation reactions occur within the lignin at low radiation dosages and stabilise the wood matrix against different processes [2,6]. Moreover, new interaction between chemical bonds can be generated in cellulose by using ionising radiation [8] and may lead to enhanced properties of wood and cellulose [11]. Through the low absorbed dose of the irradiation, the hemicelluloses (polyoses) are altered and can change the material resistance against compression [6]. Nevertheless, the influence of radiation on wood extractives has not been clarified in detail. Lawniczak and Raczkowski [12] concluded that the extractive prevents the wood for mechanical property losses caused by a radiation process.

In general, the amount of wood extractives is between 1–7% of oven dry (o.d.) weight for European softwood wood species, which is very low compared to the amount of cellulose, hemicellulose, and lignin [13]. However, wood extractives influence different wood properties (e.g., colour and durability) [14] and contain different groups of compounds (e.g., polyphenols and sugars) [15]. Overall, it could be concluded that these various groups may be affected differently by the ionising radiation based on their chemical structure.

In this study, the qualitative and quantitative chemical differences of low doses of EBI and γ-ray on larch sapwood samples were analysed using a gas chromatography–mass spectrometry (GC-MS) method. New insight into the effects of two ionising radiation sources on wood performances will help to understand the significance of the extractives. Furthermore, FT-IR vibrational spectroscopy methods were applied for the ratio assessment of lignin to cellulose within the samples to characterise the possible changes within the two wood components. 

## 2. Materials and Methods

### 2.1. Material

Seventy-five samples were obtained from larch sapwood (*Larix decidua* (Mill)), which were cut to dimensions of 1.0 × 1.0 × 1.0 cm^3^. The samples were divided into three groups of 25 pieces for the various treatments and chemical characterisation after the different irradiations. Before testing, the samples were ground with a cutting mill (Retsch, Haan, Germany) using solid carbon dioxide to pass a mesh of 500 µm. The larch wood powder was then freeze-dried to prevent chemical changes in material during drying with higher temperature.

### 2.2. Radiations

The EBI treatment was performed under normal air environmental conditions at a dose of 10 kGy with a Rhodotron TT-100 10 MeV electron accelerator (IBA International, Louvain-La-Neuve, Belgium, radiation energy of 10 MeV). The γ-ray irradiation was done in a Gammatron 1500 gamma irradiator (Mediscan, Seibersdorf, Austria) at a dose of 10 kGy by using the decay of ^60^Co.

### 2.3. Solid-Liquid Extraction

The extraction of the wood was done with an accelerated solvent extractor (ASE, Dionex Corporation, Sunnycale, CA, USA.) according to Willför et al. [16]. The larch sapwood samples were first extracted with hexane (solvent temperature 90 °C, pressure 13.8 Mpa, 2 × 5 min static cycles) and then with acetone and water in a ratio 95/5 mixture (100 °C, 13.8 Mpa, 2 × 5 min).

### 2.4. GC-MS Characterisation

Before GC-MS characterisation, different extractives were evaporated using nitrogen gas and silylated to enhance volatility. This GC-MS method was developed according to Wagner et al. [17]. For silylation, the evaporated extractives were first dried in a vacuum oven at 40 °C, and the silylated solvent (80 µL bis(trimethylsilyl)-trifluoracetamide, 20 µL pyridine and 20 µL trimethylsilyl-chloride) were added. After that, the samples were incubated at 70 °C for 45 min. Measurements were performed using a Perkin Elmer Auto-System CL gas chromatograph (PerkinElmer Inc., Waltham, MA, USA) and an MS device. The GC was equipped with an HP-5 column (length: 25 cm; ID: 0.20 mm; film thickness 0.11 µm) and a flame ionization detector (FID, Agilent Technologies Inc., Santa Clara, CA, USA). The carrying gas was nitrogen at a flow rate of 0.8 mL/min. Moreover, other conditions were: internal oven 120 °C with an increasing rate at 6 °C/min to 320 °C (15 min hold); a split injection with a ratio of 25:1 and a temperature of 250 °C; the detector temperature 310 °C and injection value of 1 µL. The data were investigated based on the mass spectra library created at the Laboratory of Natural Materials Technology at Åbo Akademic University in Turku.

### 2.5. FT-IR Spectroscopy Analysis

The obtained samples from sapwood of larch were characterised by infrared spectroscopy with a Perkin Elmer FT-IR spectrometer (Waltham, MA, USA) equipped with a Miracle diamond ATR accessory with a 1.8 mm round crystal surface. The spectra were recorded in the wavenumber range between 600 cm^−1^ and 4000 cm^−1^ with 32 scans at a resolution of 4 cm^−1^. All spectra were ATR and baseline corrected in the wavenumber range between 600 cm^−1^ and 4000 cm^−1^ as well as normalised the absorbance values between 0 and 1.

## 3. Results and Discussion

**Figure 1:**
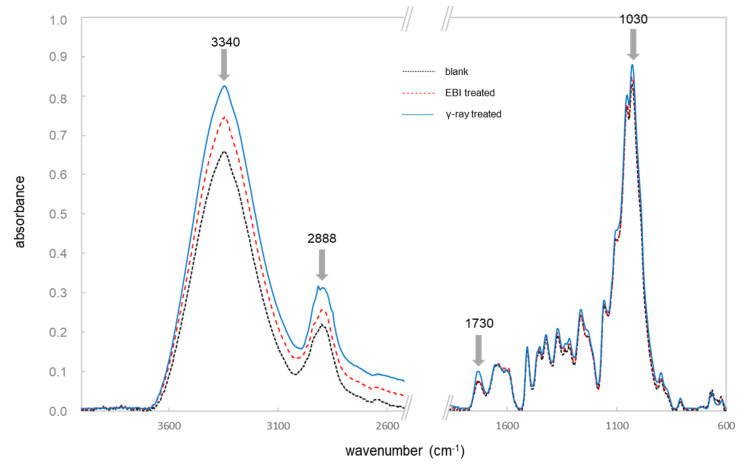
Fourier-transform infrared spectroscopy (FT-IR) spectra of larch sapwood samples with different treatments.

The FT-IR spectra from larch sapwood samples without and after different irradiation processes are presented in Figure 1. The four marked band areas show an increase in absorbance resulting from the EBI and γ-ray treated samples. The peaks obtained at 3340 cm^−1^ are an indication of intramolecular hydrogen bond of cellulose [18]. The IR signal in the range between 2933 cm^−1^ and 2835 cm^−1^ corresponded with the asymmetric CH_2_ valence vibration and the CH_2_, CH_2_OH groups in the wood material. Furthermore, the absorbance of the carbonyl groups at 1730 cm^−1^ wavenumbers changes due to irradiation.

The band at 1030 cm^−1^ corresponds to C-O valence vibration and C_alky_-O ether vibrations of different wood compounds. All these observations show that there is the separation of new molecule types from radical formation, ring, and chain breaking of the wood, mainly from the carbohydrates, which has amount around 67.1% in dry larch wood [13] and has the most important impact on the IR signals. Su et al. [19] mentioned the cellulose and lignin contents of gamma-irradiated bamboo were stable up to 300 kGy, whereas polyoses and extractives were not analysed and could protect the cellulose and lignin from degradation. This behaviour seemed reasonable, as Yang et al. [20] concluded that the cellulose from paper grade bamboo pulp were easily degraded by a ^60^Co γ-ray radiation. According to Schwanninger et al. [18], the bands around 1510 and 898 cm^−1^ correspond to the lignin and cellulose polymers, respectively. This relative ratio of these IR bands was used to assess the relative lignin content in the wood and its change [7]. Based on this approach, the results of the ratio are 1.88, 1.83, and 1.66 for untreated, EBI, and γ-ray-treated larch wood powder, respectively. While the blank and EBI-treated samples show a similar ratio, the γ-ray-irradiated sample was different and depicts a lower amount of lignin content compared to the others. Based on detailed analysis results that the absorbance of the IR band at around 898 cm^−1^ was higher than the both other sample groups, it can be assumed that the functional group of the carbohydrates increased due to the γ-ray treatment more than the absorbance of the aromatic skeletal vibration at 1510 cm^−1^.

The extracts were analysed quantitatively after the different treatments in the first steps. The extraction yields of o.d. larch sapwood with hexane differed in a range from 2.27 mg/g of blank to 1.36 mg/g of EBI treated and 1.67 mg/g of γ-ray treated samples, respectively. Through the ionising radiations, the amount of hexane extractable compounds decreased significantly, at which the EBI had the significant effect of the extracts compared to the γ-ray process. Overall, the resin acids appeared to be very sensitive to the irradiation treatments and exhibited the strongest decrease compared to the other og compound studied (Table 1). Based on the detailed analysis of single substances of the blank extracts, EBI and γ-ray-treated samples showed that almost all amounts of single compounds decreased, except palmitic acid, for which values increased for the extract from γ-ray-treated samples. On the one hand, the extractives could be cross-linked to higher molecule mass and/or other wood components resulting from the insolubility of the hexane solvent. On the other hand, the effects of the breaking of chemical bonds of wood extractives may change the compound solubility [21]. The formation of free molecules and broken glucose rings indicate the damage of cellulose. These chemical intermediates can react with the oxygen in the air and result in the change of polarity of components.

This process could be also seen by using other solvents that are more polar than hexane. Therefore, acetone was used as second solvent to recover polar compounds from the larch sapwood after different treatments. The extraction yields of o.d. larch sapwood with hexane differed in a range from 9.73 mg/g of blank to 4.48 mg/g of EBI treated and 4.80 mg/g of γ-ray treated samples, respectively. Generally, the amount of extract with acetone is higher comparted to the hexane solvent. Nevertheless, the ratio between the different treatments is quite uniform, the EBI- and γ-ray-treated samples showed less extraction yield than the blank samples. Almost all amounts of component groups increased due to the different treatment without the exception of the polyphenol groups (Table 2)—that extraction yield decreased very strongly.

Especially, dihydrokaempferol, (+)-catechin, and taxifolin from the flavonoid group were mostly affected due to the irradiation treatments. These compounds are known as antioxidants for inhibiting free radicals based on different activities (e.g., hydroxyl substituents, peroxyl reaction) in materials [22]. In the extracts of the irradiated samples, a modified taxifolin structure was quantitatively identified, which was not found in the blank samples and supports the idea of radical scavenging of flavonoids and further cross-linking effects. Also, Błaszak et al. [23] determined a high degradation rate of the flavan-3-ol substance of (+)-catechin due to 10 kGy EB irradiation compared to the gallic acid (phenolic acid), which was not affected by the EBI treatment up to this absorbed dose. The hydroxybenzoic acids like e.g., vanillic acid are possible metabolites of the phenylpropanoid pathway, and the extraction amount of group of phenylpropanoid was stable for the blank samples and the EBI-treated samples and increased strongly for the γ-ray-treated samples. The acetone extracted compounds with one or two guaiacyl units in its molecular structure were significantly increased in quantity, whereas the component amount of benzene-1,2-dicarbocylic acid with two aliphatic rest in the structure decreased from 0.164 to 0.038 mg/g by the two irradiation treatments. It can be accepted that the electrons or γ-ray starts the degradation of the aliphatic structure and some of these damaged compounds arranged to guaiacyl derivates with one or two phenyl groups.

Compared to the polyphenol groups, the amount of polyhydric alcohols increased due to the EBI and even more for the γ-ray irradiation. Cyclitols like pinitol (3-*O*-methyl-chiro-inositol) and myo-inositol (1,2,3,4,5,6-Hexahydroxycyclohexane) were found in a larger amount after irradiations than before it. Also, further sugar alcohols were determined after the ionising radiation. All these various compounds show a carbohydrate structure, and it can be assumed that these products were generated from the cellulose and polyoses by the irradiation processes.

Furthermore, two new substances were found in the extracts of larch sapwood samples after EBI or γ-ray treatments. The lignan α-conidendric acid was characterised by the extracts from both treatments, and isohydroxymatairesinol (iso-HMR) was identified only in the extract from the γ-ray treated samples. Both of these molecules have two phenyl rings within the structure that can be arranged from other polyphenols or lignans, which was shown from the decreased in polyphenols contents in the extracts. Zule et al. [24] mentioned the secoisolariciresinol was the dominantly substances of lignan in larch heartwood; however, larch sapwood was not investigated in the same study. Nevertheless, Nisula [25] concluded that sapwood contained only traces of lignan compared to the concentration in larch heartwood without giving some values. Also, the results in this study indicate that secisolariciresinol is not present in high amounts compared to the other polyphenols, such as dihydrokaempferol or taxifolin, at least in these materials which were characterised. However, the lignans were detectable and show the effects of the irradiation treatments.

## 4. Conclusions

Various chemical changes in larch sapwood extractives due to different ionization irradiation of electron beam and γ-ray technologies were observed by a FT-IR vibrational spectroscopy and GC-MS methods. The extraction yields of the samples were significantly reduced after the different treatments comparted to the unirradiated ones.

This lower amount of leaching out of wood extractives of irradiated samples could stabilise the material properties against different influences, such as weathering effects or microorganism attack. The quantitative most important substances of resin acids and polyphenols groups were highly affected of the two irradiation processes. Furthermore, two new substances were found in the extracts of larch sapwood samples after EBI or γ-ray treatments.

These results demonstrate the importance of the wood extractives on wood treatment by two ionising irradiation processes, which might have an impact on material performance.

The findings show that the different ionising radiation sources could be used as an interesting technique for changing the chemical compounds in wood.

## Figures and Tables

**Table 1:** Main component groups in hexane extracts of different treated larch sapwood samples by gas chromatography–mass spectrometry (GC-MS).

Component Groups	Blank Larch Sapwood (mg/g)	EBI-Treated Larch Sapwood (mg/g)	X-ray-Treated Larch Sapwood (mg/g)
Terpenoid	0.185	0.038	0.104
Alcohol	0.009	0.001	0.003
Fatty acids	0.486	0.124	0.217
Stilbenoid	0.005	0.000	0.000
Resin acids	2.083	0.177	0.547
Aliphatic compounds	0.005	0.001	0.002
Hydroxy resin acids	0.051	0.010	0.023
Lignans	0.062	0.002	0.011
Precursor of Lignin	0.020	0.005	0.010
Unknown	0.962	0.130	0.306

**Table 2:** Main component groups in acetone extracts of different treated larch sapwood samples by GC-MS.

Component Groups	Blank Larch Sapwood (mg/g)	EBI-Treated Larch Sapwood (mg/g)	X-ray-Treated Larch Sapwood (mg/g)
Carboxylic acids	0.051	0.155	0.348
Phenylpropanoid	0.242	0.253	0.654
Polyhydric alcohols	0.027	0.195	0.486
Single sugars	0.415	0.832	2.043
Aliphatic acids	0.064	0.096	0.323
Resin acids	0.028	0.061	0.225
Polyphenols	7.019	0.499	2.659
Phytosterine	0.028	0.005	0.019

## References

[1] Saeman J.F., Millett M.A., Lawton E.J. (1952). Effect of high-energy cathode rays on cellulose. Ind. Eng. Chem..

[2] Fischer K., Goldberg W. (1987). Changes in lignin and cellulose by irradiation. Makromol. Chem. Marcomol. Symp..

[3] Buremester A. (1967). The improvement of wood by radiation-initiated polymerisation of monomer plastic. Holz Roh Werkst..

[4] Seifert K. (1964). Zur Chemie gammabestrahlten Holzes. Holz Roh Werkst..

[5] Hoffmann P., Schweers W. (1976). On the hydrogenolysis of lignin. 10. Comparative hydrogenolyses of lignins, lignosulfonic acid, and lignosulfonate model compounds under irradiation with γ-rays. Paperi Ja Puu..

[6] Schnabel T., Huber H., Grünewald T., Lichtenegger H.C., Petutschnigg A. (2015). Changes in mechanical and chemical wood properties by electron beam irradiation. Appl. Surf. Sci..

[7] Schnabel T., Huber H. (2014). Improving the weathering on larch wood samples by electron beam irradiation (EBI). Holzforschung.

[8] Baccaro S., Carewska M., Casieri C., Cemmi A., Lepore A. (2013). Structure modifications and interaction with moisture in γ-irradiated pure cellulose by thermal analysis and infrared spectroscopy. Polym. Degrad. Stabil..

[9] LaVerne J.A., Driscoll M.S., Al-Sheikhly M. (2000). Radiation stability of lignocellulosic material compounds. Radiat. Phys. Chem..

[10] Huber H., Haas R., Petutschnigg A., Grüll G., Schnabel T. (2020). Changes in wettability of wood surface using electron beam irradiation. Wood Mater. Sci. Eng..

[11] Henniges U., Hasani M., Potthast A., Westman G., Rosenau T. (2013). Electron beam irradiation of cellulosic materials—Opportunities and limitations. Materials.

[12] Lawniczak M., Razkowski J. (1970). The influence of extractives on the radiation stability of wood. Wood Sci. Technol..

[13] Fengel D., Grosser D. (1975). Chemical composition of softwoods and hardwoods—A bibliographical review. Holz Roh Werkst..

[14] Fengel D., Wegner G. (2003). Wood Chemistry Ultrastructure Reactions.

[15] Wagner K., Roth C., Willför S., Musso M., Petutschigg A., Oostingh G.J., Schnabel T. (2019). Identification of antimicrobial compounds in different hydrophilic larch bark extracts. BioRescources.

[16] Willför S.M., Hemming J., Raunanen M., Holmbom B. (2003). Phenolic and lipophilic extractives in Scots pine knots and stemwood. Holzforschung.

[17] Wagner K., Musso M., Kain S., Willför S., Petutschnigg A., Schnabel T. (2020). Larch wood residues valorization through extraction and utilization of high value-added products. Polymers.

[18] Schwanninger M., Rodrigues J.C., Pereira H., Hinterstoisser B. (2004). Effects of short-time vibratory ball milling on the shape of FT-IR of wood and cellulose. Vib. Spectrosc..

[19] Su F., Jiang J., Sun O., Lu F. (2014). Changes in chemical composition and microstructure of bamboo after gamma ray irradiation. BioRescources.

[20] Yang G., Zhang Y., Wei M., Shao H., Hu X. (2010). Influence of γ-ray radiation on the structure and properties of paper grade bamboo pulp. Carbohydr. Polym..

[21] Polvi J., Nordlund K. (2014). Low-energy irradiation effects in cellulose. J. Appl. Phys..

[22] Heim K.E., Tagilferr A.R., Bobilya D.J. (2002). Flavonoid antioxidants: Chemistry, metabolism and structure-activity relationships. J. Nutr. Biochem..

[23] Błaszak M., Nowak A., Lachowicz S., Migdał W., Ochmian I. (2019). E-Beam irradiation and ozonation as an alternative to the sulphuric method of wine preservation. Molecules.

[24] Zule J., Čufar K., Tišler V. (2016). Hydrophilic extractives in heartwood of European larch (*Larix decidua* Mill). Drv. Ind..

[25] Nisula L. (2018). Wood Extractives in Conifers. A Study of Steamwood and Knots of Industrially Important Species. Ph.D. Thesis.

